# A prospective study on body image disturbances during pregnancy and postpartum: the role of cognitive reappraisal

**DOI:** 10.3389/fpsyg.2023.1200819

**Published:** 2023-08-09

**Authors:** Marta Spinoni, Claudio Singh Solorzano, Caterina Grano

**Affiliations:** Department of Psychology, Sapienza University, Via dei Marsi, Rome, Italy

**Keywords:** pregnancy, emotion regulation strategies, body image disturbances, cognitive reappraisal, postpartum body image

## Abstract

**Background:**

During pregnancy, body size rapidly modifies over a relatively short period. Literature emphasizes the need to identify the factors that influence body image during peripartum as the extent of women’s adaptation to these changes has significant repercussions on both mother’s and newborn’s health. Emotion regulation strategies (i.e., expressive suppression and cognitive reappraisal) were linked to body image in the general and clinical population, but no studies were conducted in the peripartum. The present study aims to investigate the longitudinal impact of prepartum body image disturbances on postpartum body image disturbances and to evaluate the mediational role of emotional regulation strategies.

**Methods:**

A total of 133 pregnant women completed a three-phase longitudinal study. Women answered online questionnaires during the second (T1) and the third (T2) trimesters of pregnancy, and at about 6 months postpartum (T3).

**Results:**

Findings indicated that body image disturbances at T1 were a significant predictor of body image disturbances in the postpartum (1 year after the first assessment). Moreover, cognitive reappraisal measured at T2 partially mediated this relationship: body image disturbances in the second trimester of pregnancy were linked to less use of cognitive reappraisal in the third trimester of pregnancy, and this, in turn, was associated with worse body image disturbances at 6 months after birth.

**Conclusion:**

Findings of this longitudinal study highlight the importance of assessing body image disturbances during pregnancy to early identify women at risk, and suggest cognitive reappraisal as a possible target intervention.

## 1. Introduction

Body image disturbance (or “negative body image”) is a multidimensional construct, that refers to negative perceptions, feelings, behaviors, beliefs, and attitudes toward aspects of one’s body (Cash et al., 2004). While body dissatisfaction mainly concerns negative feelings about one’s body, body image disturbance is defined by a negative evaluation of one’s appearance not only on emotional terms but also on perceptual, cognitive, and behavioral ones. A negative body image includes preoccupation and worries related to weight or body aspects including the resulting interference with daily life or functioning (Phillips, 2005).

During pregnancy, body size and weight rapidly change over a relatively short period, leading the woman to move away from the socio-culturally prescribed “thin ideal” and triggering additional body image concerns (Skouteris et al., 2005; Duncombe et al., 2008; Watson et al., 2015). Although the normal societal pressure to conform to body shape ideals may be reduced during pregnancy (Duncombe et al., 2008), several studies reported an increase in body dissatisfaction during the peripartum period (Fuller-Tyszkiewicz et al., 2013), with further intensifications in the postpartum (Hodgkinson et al., 2014; Tavakoli et al., 2021). Indeed, during the postpartum period, most women report more concerns about their inability to return to their pre-pregnancy body weight, resulting in higher body dissatisfaction (Lovering et al., 2018; Nagl et al., 2021).

The extent of pregnant women’s adaptation to their changing bodies has significant implications. Body image disturbances during pregnancy significantly increase the risk of developing perinatal depression (Silveira et al., 2015; Riquin et al., 2019), with subsequent negative implications on mother–child interactions and caretaking activities (Slomian et al., 2019). Negative body image is associated with problematic parent–child feeding interactions (Bergmeier et al., 2020), with lower breastfeeding intentions (Barnes et al., 1997), and poorer mother-fetal and mother-newborn attachment quality (Huang et al., 2004; Williams, 2019). Furthermore, during the peripartum, dissatisfaction with one’s body is associated with higher rates of unhealthy behaviors, like skipping meals, self-induced vomiting, and laxative use as extreme weight control conducts (Clark and Ogden, 1999; Neiterman and Fox, 2017). Besides being detrimental to the women, these unhealthy dieting and dysfunctional conducts are also harmful to the fetus and the baby’s development (Chan et al., 2020).

Not all women report an increase in worries and concerns about their bodies during pregnancy, and psychosocial characteristics may mediate this relationship. Indeed, studies highlighted that some pregnant women are even more satisfied with their bodies than non-pregnant women (Richardson, 1990; Loth et al., 2012). For this reason, the literature emphasizes the need to identify the factors that differently contribute to negative body image (Sweeney and Fingerhut, 2013; Watson et al., 2015).

In the non-pregnant population, researchers studied the role of emotion regulation strategies, evidencing positive associations between emotional regulation difficulties and body image (i.e., Hayaki et al., 2002; Sim and Zeman, 2006; Hughes and Gullone, 2011). Two of the most explored emotional regulation strategies are cognitive reappraisal and expressive suppression. *Cognitive reappraisal* is an antecedent-focused strategy that consists of the cognitive change of the emotional impact of a situation; it is considered a positive strategy consisting in the reinterpretation of a stressor with an effective alteration of its emotional trajectory, which predicts lower negative affect (Gross and John, 2003; Aldao et al., 2010). In contrast, *expressive suppression* is the inhibition of the already-formed emotional response (Gross and John, 2003; Gross and Thompson, 2007; Gross, 2015). As a response-focused strategy that comes late in the emotion-generative process, suppression of emotions is considered an inefficient strategy, creating a discrepancy between the inner experience and the outer expression of emotion (Gross and John, 2003). It is associated with more negative feelings, lower levels of self-esteem, and worst relationships with others (Gross, 1998; McLean et al., 2007; Berking and Wupperman, 2012; Kraiss et al., 2020).

Studies on body image highlighted that subjects with greater use of expressive suppression report higher levels of negative body image than those who use cognitive reappraisal as a usual strategy (Geller et al., 2000; Hughes and Gullone, 2011; Kiriukhina and Polskaya, 2021). Conversely, cognitive reappraisal strategies are associated with higher body image satisfaction, less shape, and eating concerns, with greater acceptance of physical appearance changes, and with more frequent adoption of healthy behaviors (Siân et al., 2010; Prefit et al., 2020). Literature has also evidenced that a negative body image is related to increased cognitive arousal, higher distress, more negative thoughts, and reduced use of adaptive emotion regulation strategies (Cash et al., 2005; Sim and Zeman, 2005; Ferreira and Trindade, 2015). Moreover, in the non-pregnant population, it is well documented that a reduced ability to properly regulate one’s feeling through adaptive emotional regulation strategies may lead to different expressions of psychopathology (Lappanen et al., 2022; Miu et al., 2022; Stellern et al., 2023). However, as recently pointed out by Penner and Rutherford (2022), there is limited research evaluating the role of emotion regulation on women’s mental health throughout the perinatal period (Penner and Rutherford, 2022). This is surprising, considering that pregnancy is a period full of physiological and emotional changes, in which emotion regulation strategies may play a crucial role in reducing or exacerbating the distress or the risk of psychopathology (Rutherford et al., 2015; Coo et al., 2020; McDonald et al., 2021; Taubman-Ben-Ari et al., 2021). Considering body image, to the best of our knowledge, no studies evaluated the relationship of emotional regulation with body image disturbances during the peripartum period.

Based on the above considerations, we hypothesize that women reporting more body image disturbances during pregnancy will be at greater risk of postpartum body image disturbances. Moreover, we aim to examine the mediating role of emotional regulation strategies in this relationship. In particular, we assume that prepartum body image disturbances will be negatively associated with the use of cognitive reappraisal and that this in turn will negatively predict body image disturbances in the postpartum. Conversely, we expect that negative body image in the second trimester of pregnancy will be positively associated with the use of expressive suppression in the third trimester of pregnancy, which, in turn, will positively predict body image disturbances at 6 months after birth.

## 2. Materials and methods

### 2.1. Participants and procedures

Two hundred and thirty healthy pregnant women accepted to participate in a three-phase longitudinal study. Women were directly and opportunistically recruited through private maternity centers, schools, family associations, and diagnostic clinics in Italy between February 2018 and October 2020. Inclusion was limited to women in the second trimester of pregnancy (the fourth, fifth, or sixth month) and who were fluent in Italian. We choose not to include women in the first trimester as body changes are not so evident in this period. All participants took part on a voluntary basis and were not remunerated. After explaining the procedure of the study and signing the informed consent form, women in the second semester of pregnancy were asked to complete an online questionnaire (T1). Participants were required to provide an email address and a phone number for further contact. During the third trimester and after the baby was 6 months old, participants were contacted again by email or phone message and asked to complete a second (T2) and a third (T3) online questionnaire. To guarantee participants’ anonymity, a personal alphanumeric code was assigned to each participant and used to access the online questionnaires and implemented through the survey editor SurveyMonkey. Out of the 230 pregnant women who completed valid baseline measurements, participants included in the final analyses were the 133 women with complete data for all variables at baseline and follow-ups, including covariates. All participants gave fully informed written consent to participate in the study, and ethical approval was obtained by the Institutional Review Board of the Psychology Department, Sapienza University of Rome (Prot. n. 0000102 of 24/01/2018).

### 2.2. Measures

#### 2.2.1. Body image disturbance

The Body Image Disturbance Questionnaire (BIDQ) was used to assess body image disturbance or “negative body image” (Cash et al., 2004; Cash and Grasso, 2005) at T1, T2, and T3. Permission to use the questionnaire was obtained by the authors. The seven items of the scale explored concerns, preoccupations, distress, and impairment in social or occupational functioning related to participants’ body appearance (Cash, 2002; Cash and Grasso, 2005). An example item is: “*Are you concerned about the appearance of some part(s) of your body which you consider especially unattractive?*” Responses to each statement are scored on a five-point Likert scale ranging from 1 to 5. The total score is taken as the mean of the seven items, with a higher score indicating higher levels of body image disturbance (Cash et al., 2004). The scale showed excellent internal consistency and test–retest reliability (Cash and Grasso, 2005; Hrabosky et al., 2009) and was used in previous studies on pregnant women (Shloim et al., 2019). The BIDQ was translated into Italian. A factor analysis using Principal Axis Factoring method was applied to the T1 BIDQ responses to verify the one-factor structure of the questionnaire The values of the Kaiser–Meyer–Olkin (KMO) of 0.822 and the result of Bartlett’s test [χ^2^(21) = 388.23, *p* < 0.001] indicated the suitability of the data for factor analysis (Hutcheson and Sofroniou, 1999). The Italian version of BIDQ proved to be one-dimensional, as indicated by the total variance explained by this factor (50.01%) and the fact that all the seven items of the BIDQ loaded onto this factor and had good to excellent factor loadings (range: 0.55–0.81) (Tabachnick and Fidell, 2013). In the current sample, Cronbach’s alphas were α = 0.82 (T1), α = 0.87 (T2), and α = 0.89 (T3) and McDonald’s omega were ω = 0.81 (T1), ω = 0.86 (T2), and ω = 0.88 (T3). To further explore the change over time of the variable, we calculate a change score (i.e., subtraction of T1 Body Image Disturbance from T3 Body Image Disturbance). Positive scores indicated an increase in body image disturbance over time.

#### 2.2.2. Emotion regulation

Emotion regulation was evaluated at T2 using the Italian version of the Emotion Regulation Questionnaire (ERQ; Gross and John, 2003; Balzarotti et al., 2010). The 10-item scale measures participants’ typical use of cognitive reappraisal and expressive suppression as strategies to regulate emotional responses (Gross and John, 2003; Gross, 2015). Each item is scored on a seven-point Likert scale ranging from 1 (strongly disagree) to 7 (strongly agree). The questionnaire provides two mean scores related to the six-item cognitive reappraisal subscale (ERQ-R) and the four-item expressive suppression subscale (ERQ-S). Two examples of items are “I control my emotions by changing the way I think about the situation I’m in” for the Cognitive Reappraisal subscale, and “I control my emotions by not expressing them” for the Expressive Suppression Subscale. Greater scores at each subscale indicate greater emotion regulation strategy use. The scale showed good factorial validity and internal consistency reliability (Balzarotti et al., 2010; Preece et al., 2020, 2021) and was used in recent peripartum research (Zemestani and Fazeli Nikoo, 2020; Taubman-Ben-Ari et al., 2021). Cronbach’s alpha for the ERQ-R and ERQ-S subscales at T2 was α = 0.89 and α = 0.75, respectively. McDonald’s omega for the ERQ-R and ERQ-S subscales at T2 were 0.90 and 0.76, respectively.

#### 2.2.3. Covariates

Participants’ age, educational levels, and parity were recorded by the T1 self-report questionnaire. The delivery method was recorded at postpartum assessment. Height was measured at T1, and weight was measured at each phase of the study (i.e., T1, T2, and T3). BMI was calculated using the standard formula (kg/m^2^). To explore the change over time of BMI, we also calculate two change scores (i.e., subtraction of T1 BMI from T3 BMI and subtraction of T1 BMI from T2 BMI). Positive scores indicated an increase in BMI. Depressive symptoms at T1 were assessed using the Italian version of the Edinburgh Postnatal Depression Scale (EDPS; Cox et al., 1987; Benvenuti et al., 1999). This instrument evaluates depressive symptoms in the previous week using a four-point Likert scale. Total scores ranged from 0 to 30, with higher scores indicating a greater frequency of depressive symptoms. The EPDS has been shown to be a reliable instrument for screening depressive symptoms in the peripartum period (Bergink et al., 2011; Levis et al., 2020) with a clinical cut-off of 12 in the Italian population (Benvenuti et al., 1999). In the current study, Cronbach’s alpha was α = 0.79 and McDonald’s omega was 0.80.

### 2.3. Statistical analyses

Data analyses were conducted using IBM SPSS Statistic version 28 (SPSS Inc.). Sensitivity analyses were carried out to check for significant differences in the characteristics of participants between those who only completed the first assessment (T1) and those with full data. Using independent *t* and *χ*^2^ tests, women were compared on age, education, parity, BMI, prepartum depressive symptoms, and prepartum body image disturbance. Descriptive statistics were calculated for each variable of the study. Means (M), standard deviations (SD), and min/max range were presented for all continuous variables. Data on categorical variables were presented as the number of participants (N) and percentages. Paired *t*-test was used to explore the change in body image disturbance from the prepartum to the postpartum period. Pearson’s product–moment correlation coefficients (*r*) were calculated to explore the relations among covariates, T1 depressive symptoms, T1 body image disturbances, T2 cognitive reappraisal, T2 expressive suppression, T3 body image disturbances, change scores in BMI from T1 to T3, and change scores in BIDQ from T1 to T3. The effect size of absolute value of *r* was considered small (0.10), medium (0.30), or large (0.50; Cohen, 1988). To examine whether emotion regulation would mediate the relationship between prepartum and postpartum body image disturbance, bootstrap mediation analysis for simple mediation through model 4 of SPSS PROCESS macro 4.0 was applied (Preacher and Hayes, 2008; Hayes, 2017). The method included 5,000 bootstrap samples for coefficient and indirect estimation and 95% bias-corrected confidence intervals (CI) for the indirect effect.

To minimize the potential bias of the simultaneous measurement of two variables, we considered each variable of the mediation model at a different time point.

Change scores in BMI (T3-T1) was entered as covariate. We reported the unstandardized effect size (b) and the 95% bias-corrected CI for the indirect effect model. If the 95% CI does not include zero, the mediation effect is significant.

## 3. Results

The participation rate in the entire study is 57.8%. Compared to the women who completed all the phases of the study (*N* = 133), those who did not complete the second and third assessments (*N* = 97) were more likely to have high levels of depressive symptoms at T1 [*t*(228) = 2.452; *p =* 0.015]. There were no other significance differences: age [*t*(228) = 0.678; *p =* 0.499], education [χ^2^(5) = 9.293, *p* = 0.098], BMI [*t*(228) = 0.307; *p =* 0.759], and body image disturbance at T1 [*t*(228) = −0.711; *p =* 0.478].

Table 1 shows the demographic characteristics of the sample. The average age of the sample was 32.09 (SD = 4.83) years, with an age range between 20 and 42. More than half of the participants had at least a university education (58.6%), were primiparous (59.4%), and had a vaginal delivery (68.4%).

**Table 1:** Descriptive characteristics of participants (*N* = 133).

Variable	Mean ± SD (min – max) or *N* (%)
Age	32.09 ± 4.83 (20–42)
Education	
Middle school	7 (5.3)
High school	48 (36.1)
Bachelor	25 (18.8)
Master’s degree	36 (27.1)
Post lauream specialization courses	7 (5.3)
PhD	10 (7.4)
Month of pregnancy (T1)	
4th	61 (45.9)
5th	57 (42.9)
6th	15 (11.3)
Month of pregnancy (T2)	
7th	41 (30.8)
8th	87 (65.4)
9th	2 (1.5)
10th	3 (2.3)
Parity status	
Primiparous	79 (59.4)
Multiparous	54 (49.6)
Delivery method	
Spontaneous vaginal delivery	64 (48.1)
Induced vaginal delivery	27 (20.3)
Elective cesarean section	25 (18.8)
Emergency cesarean section	17 (12.8)
BMI	
T1	24.99 ± 4.47 (15.98–40.01)
T2	26.92 ± 4.15 (17.58–41.02)
T3	25.07 ± 5.10 (16.16–40.04)
Depressive symptoms (T1)	6.71 ± 4.27 (0–21)
Body image disturbance (T1)	1.72 ± 0.53 (1–3.57)
Cognitive reappraisal (T2)	5.07 ± 1.31 (1–7)
Expressive suppression (T2)	2.94 ± 1.16 (1–7)
Body image disturbance (T2)	1.65 ± 0.56 (1–3.86)
Body image disturbance (T3)	1.75 ± 0.62 (1–3.57)
Change score in BMI (T2-T1)	1.92 ± 1.26 (−1.47–6.48)
Change score in BMI (T3-T1)	0.08 ± 2.75 (−4.69–15.01)
Change score in body image disturbance (T3-T1)	0.03 ± 0.48 (−1.43–1.29)

Body image disturbance did not change significantly over the peripartum period [*t*(132) = −0.804, *p* = 0.422]. Depression scores ranged from 0 to 21, with 21.1% of women above the cut-off score of 12 in the prepartum period (T1). Correlations among the variables of interest considered are reported in Table 2. Depressive symptoms at the initial phase of pregnancy were weakly correlated with body image disturbance at the same time (*r* = 0.176, *p* = 0.042) and with the expressive suppression subscale at T2 (*r* = 0.271, *p* = 0.002). Moreover, greater levels of body image disturbance at T1 were weakly correlated with higher levels of expressive suppression at T2 (*r* = 0.191, *p* = 0.028), and moderately associated with levels of cognitive reappraisal at T2 (*r* = −0.331, *p* < 0.001) and strongly associated higher levels of body image disturbance at postpartum (*r* = 0.665, *p* < 0.001). Greater usage of cognitive reappraisal strategy was moderately correlated with lower levels of body image disturbance at T3 (*r* = −0.477, *p* < 0.001). Moreover, a greater increase in the BMI change from T1 to T3 was correlated with a higher increase in the BDI scores from T1 to T3 (*r* = 0.252, *p* = 0.003).

**Table 2:** Correlations between variables of the present study (*N* = 133).

Variable	1	2	3	4	5	6	7	8	9	10	11	12	13
1. Age	-												
2. Education	0.346^*^	-											
3. BMI (T1)	0.016	0.012	-										
4. Parity	0.306^*^	0.008	0.094	-									
5. Delivery method	0.083	0.051	0.032	−0.126	-								
6. Depressive symptoms (T1)	−0.074	−0.104	0.040	−0.040	0.002	-							
7. Body image disturbance (T1)	−0.044	−0.075	0.468^*^	0.088	0.069	0.176^^^	-						
8. ERQ-cognitive reappraisal (T2)	0.041	0.058	−0.219^*^	0.017	−0.087	−0.122	−0.331^*^	-					
9. ERQ-expressive suppression (T2)	−0.107	−0.194^^^	0.089	0.037	−0.038	0.271^*^	0.191^^^	−0.085	-				
10. Body image disturbance (T3)	−0.032	−0.062	0.418^*^	0.084	0.046	0.041	0.665^*^	−0.477^*^	0.130	.-			
11 Change score in BMI (T2–T1)	0.025	0.013	−0.387^*^	0.084	0.010	−0.082	−0.242^*^	0.087	−0.069	−0.165	-		
12. Change score in BMI (T3–T1)	−0.185^^^	−0.140	−0.078	0.031	−0.073	−0.067	−0.011	−0.107	−0.056	−0.185^^^	0.210^^^	-	
13. Change score in BIDQ (T3–T1)	0.008	0.004	0.021	0.011	−0.017	−0.143	−0.252^*^	−0.249^*^	−0.044	0.556^*^	0.056	0.252^*^	-

^*p* < 0.05, ^*^p < 0.01. BIDQ, body image disturbance questionnaire.

**Figure 1:**
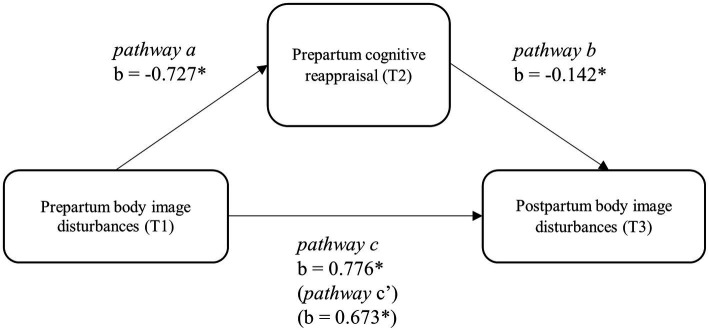
Mediation model of prepartum body image disturbance and postpartum body image disturbance through prepartum cognitive reappraisal. ^*^p < 0.05.

Given the correlation results, we examined the indirect effect of prepartum body image disturbances on postpartum body image disturbances through cognitive reappraisal levels. Change score in BMI (T3-T1) was considered as the only covariate of the path analysis, since other potential covariates were not significantly related the mediator or the outcome variable (Frigon and Laurencelle, 1993). Preacher and Hayes (2008) bootstrapping estimates of indirect effects were employed. The overall model was significant, *F*(3, 129) = 50.909, *p* < 0.001, *adj. R^2^* = 0.542. Figure 1 displays the unstandardized regression coefficients among the model variables. Higher prepartum body image disturbances were associated with lower levels of cognitive reappraisal (*b* = −0.727, 95% CI [−1.083, −0.370]). Moreover, the relationship between cognitive reappraisal and postpartum body image disturbances was significant (*b* = −0.142, 95% CI [−0.209, −0.076]). The total effect of prepartum body image disturbances on postpartum body image disturbances was significant (*b* = 0.776, 95% CI [0.630, 0.922]), as well as the direct effect of body image disturbances during pregnancy on body image after childbirth, when the cognitive reappraisal was included in the model (*b* = 0.673, 95% CI [0.527, 0.818]). The indirect effect of cognitive reappraisal at T2 in the model was significant (*b* = 0.103, SE = 0.036, 95% CI [0.043, 0.184]), indicating that higher cognitive reappraisal levels at T2 significantly and partially mediated the relationship between prepartum and postpartum body image disturbances scores.

## 4. Discussion

The present study aimed to assess body image disturbances in the peripartum period and their relationship with emotional regulation strategies. First, we hypothesized that a negative body image in the second trimester of pregnancy would affect body image disturbances evaluated 6 months after childbirth. Second, we hypothesized that emotional regulation strategies evaluated in the third trimester of pregnancy would play a mediating role in this relationship. Extending the scarce literature on the role of emotion regulation on women’s mental health throughout the perinatal period (Penner and Rutherford, 2022), the findings of the present study confirmed that prepartum body image disturbances predicted postpartum body image disturbances and proved that cognitive reappraisal partially mediated this relationship.

In the present study, pregnancy levels of body image disturbances were predictive of body image disturbances 6 months after birth. This is in line with previous longitudinal studies (Skouteris et al., 2005; Shloim et al., 2019) and is relevant as a negative body image experienced during the peripartum period may lead to negative consequences for the mother and the child (Fuller-Tyszkiewicz et al., 2013; Silveira et al., 2015; Bergmeier et al., 2022).

Regarding the main aim of the study, i.e., the mediational role of emotional regulation strategies, findings indicated that cognitive reappraisal partially mediated the relationship between body image disturbances during pregnancy and body image disturbances in the postpartum period.

Considering the pathway that links body image disturbances to cognitive reappraisal in pregnancy, our findings suggested that higher levels of body image disturbances in the second trimester of pregnancy might have influenced the use of cognitive reappraisal as an emotional regulation strategy in late pregnancy. Although our study is the first, to our knowledge, which has highlighted the association between negative body image and cognitive reappraisal in pregnant women, these findings are in line with previous studies on the non-pregnant population, in which the link between body image and emotion dysregulation was already established. Indeed, studies on the non-pregnant population showed that unsatisfaction with physical changes could affect emotional distress altering one’s body-related feelings and perceptions, resulting in more negative views and attitudes toward life (Duggan et al., 2013) and emotional regulation difficulties (Haynos and Fruzzetti, 2011; Cheng and Wei, 2022). People who experience higher discontent related to their appearance are more likely to develop emotional distress due to an inability to achieve their ideal and may use ineffective emotional regulation strategies to manage this discomfort (Sim and Zeman, 2005; Hughes and Gullone, 2011; Wilson et al., 2014). Indeed, to face these negative emotions, individuals with body image disturbance may exhibit an alteration in the representation of their bodies, may report negative thoughts about their image, exaggerated attention to their physical changes and experience different body-checking behaviors (Cash, 2002; Carey and Preston, 2019). These attitudes about one’s body can prevent individuals from focusing on internal states, leading to less awareness and acceptance of one’s emotions and to a consequent lack of access to adaptive emotion regulation strategies (Sim and Zeman, 2005; Lavender et al., 2015; Fernandes et al., 2017). A negative body image may also influence individuals’ ability to shift attention and to positively reevaluate the circumstances (Jones and Rogers, 2003; Siân et al., 2010). Previous studies in non-pregnant populations investigated the relationship between body image and other dysfunctional emotional regulation strategies, like avoidance, body-related co-rumination, and reduced levels of positive rational acceptance, and evidenced that a negative body image was significantly associated with emotion regulation difficulties (Cash et al., 2005; Ferreira and Trindade, 2015; Kiriukhina and Polskaya, 2021). Our study extends these results being the first that longitudinally evaluate the negative association of body disturbances with cognitive reappraisal in peripartum women. Different processes may explain this relationship including impaired interoceptive awareness (Füstös et al., 2013). Future studies are needed to further investigate possible mediational pathways.

Cognitive reappraisal in pregnancy was negatively associated with body image disturbances at 6 months postpartum. In previous research on the non-pregnant population, this adaptive strategy was related to a lower risk of body dissatisfaction (Siân et al., 2010; Prefit et al., 2020). Moreover, this finding is consistent with earlier research showing the importance of cognitive reappraisal in regulating stressful emotions in expecting mothers (Taubman-Ben-Ari et al., 2021). Former studies also showed that lowering thin ideals and expectations or accepting transformations in one’s body may lead to lower levels of negative body image and eating concerns (Webster and Tiggemann, 2003; Ferreira and Trindade, 2015). In this view, the process of reevaluating pregnant body shapes related to the specific moment of new motherhood may be a way to modify and adjust one’s perception, preventing the dysfunctional pressure to reach a strict pre-pregnancy thin ideal (Richardson, 1990). Although cognitive reappraisal training was effective in reducing body dissatisfaction in the general population (Fitzpatrick et al., 2019; Prefit et al., 2020), more studies are needed to evaluate whether this training may be useful also for pregnant women, especially for those who are more at risk for body image disturbances.

Finally, unlike expected, no relationship was found between expression suppression in pregnancy and body image disturbances in postpartum. In the non-pregnant population, expressive suppression is widely recognized as one of the main maladaptive emotional regulation strategies, predicting several repercussions on physical and mental health (Gross and John, 2003; Aldao et al., 2010). Moreover, previous studies reported a positive association between greater use of expressive suppression and higher levels of body dissatisfaction, evidencing that the inhibition of emotional expression is significantly correlated to negative feelings and thoughts about the body (Geller et al., 2000; Hayaki et al., 2002; Naumann et al., 2016; Kiriukhina and Polskaya, 2021). In our hypotheses, expressive suppression would have exacerbated body image disturbances in pregnancy and further maintained them in the postpartum; however, in our data, expressive suppression was only positively correlated with body image disturbances in the prepartum but not in the postpartum. It is possible that emotional suppression may be less relevant in the peripartum compared to pregnant women or women in the general population. This may be due to the fact that in the peripartum, women may experience more intense emotions compared to the general population (Pearson et al., 2009; Newham and Martin, 2013). Expression of these emotions during pregnancy is socially accepted and this may lead women to use fewer suppression strategies. Consistently, the means of expressive suppression in our sample were quite low. Future studies may confirm whether the role of expressive suppression is less relevant in the peripartum compared to other life periods.

### 4.1. Limitations

The findings of this study need to be seen in the light of some limitations. The first consideration is that all the variables were evaluated through self-report questionnaires not specifically developed to be used in pregnant women. However, all the used measures were widely used in the general, clinical, and pregnant populations. Secondly, we have to highlight the lack of a back-translation for the BIDQ, even if the translated version demonstrated adequate psychometric properties. Another limitation is the fact that we did not record the number of women who initially declined to take part in the research who may had different characteristics compared to those who consented. It must be noted, that among those who participated, women who withdrew from the study were more depressed than those who answered all the phases of the survey. Although there were no differences in body image disturbances between the women who did not participate in all the research phases and those who did, it is possible that depressed women who withdraw from the study would have had more probability of developing body image disturbances over time or that suppression would have differently affected this relationship. It has also to be noted that the levels of body image disturbances in our sample were very low, both during pregnancy and at 6 months postpartum. Moreover, in this study, we focused on negative body image. Future studies should evaluate positive aspects of body image also considering that pregnancy may be a time of relief from thinness ideals. Furthermore, we did not have a measure of cognitive reappraisal at T1. Therefore, it was not possible to control for the baseline level of cognitive reappraisal and it was not possible to measure cognitive reappraisal changes as a result of the level of body image disturbance. Similarly, we did not evaluate how emotional regulation strategies changed from pre-pregnancy to the peripartum period over time. Finally, since body image disturbances are related to depressive symptoms, future studies may need to consider the effects of the examined relationship also on postpartum depression.

## 5. Implications for practice and/or policy

Acknowledging the limitations above, we believe that the implications of our study are numerous. First, it highlights the importance of assessing women at risk for body image disturbances in the early stages of pregnancy. Second, since the positive re-evaluation of one’s pregnancy-related body changes can protect from developing body image disturbances in the postpartum, cognitive reappraisal as a key intervention target may be appropriate, especially for those women who are more at risk. Future studies with clinical trials are needed to evaluate whether implementing interventions based on adaptive emotional regulation strategies may be effective in protecting from body image disturbances in the postpartum period.

## 6. Conclusion

In conclusion, our findings suggested that prepartum body image disturbances predicted postpartum body image disturbances and proved that cognitive reappraisal partially mediated this relationship. The present study has several strengths. While most of the research on body image in pregnancy used cross-sectional designs, the longitudinal design of the present study allowed us to evidence the significant association between negative body image during pregnancy and body image disturbances in the postpartum. Moreover, the mediational model highlighted the importance of considering cognitive reappraisal as an adaptive emotion regulation strategy implicated in the relationship between prenatal and postpartum body image disturbances. As body image also had a role in influencing cognitive reappraisal strategies, more studies on this link are foreseen.

## Data availability statement

The datasets presented in this article are not readily available because the raw data supporting the conclusions of this article will not be made available by the authors. Requests to access the datasets should be directed to Caterina.grano@uniroma1.it.

## Ethics statement

The studies involving humans were approved by Institutional Review Board of the Psychology Department, Sapienza University of Rome (Prot. n. 0000102 of 24/01/2018). The studies were conducted in accordance with the local legislation and institutional requirements. The participants provided their written informed consent to participate in this study.

## Author contributions

CG conceived the study and revised the manuscript. CS and CG analyzed data. MS wrote a first draft. All authors contributed to the article and approved the submitted version.

## Funding

This work was supported by PRIN grant (Progetti di Ricerca di Rilevante Interesse Nazionale, Edit. 2017, Prot. n. 2017BC4MST) to CG.

## Conflict of interest

The authors declare that the research was conducted in the absence of any commercial or financial relationships that could be construed as a potential conflict of interest.

## Publisher’s note

All claims expressed in this article are solely those of the authors and do not necessarily represent those of their affiliated organizations, or those of the publisher, the editors and the reviewers. Any product that may be evaluated in this article, or claim that may be made by its manufacturer, is not guaranteed or endorsed by the publisher.
